# Forearm Compartment Syndrome

**Published:** 2014-04-23

**Authors:** Jeon Cha, Blair York, John Tawfik

**Affiliations:** The Sydney Hospital Hand Unit. Sydney Hospital and Sydney Eye Hospital, Sydney, Australia

**Keywords:** compartment syndrome, fasciotomy, forearm compartments, forearm anatomy, upper limb ischaemia

## DESCRIPTION

A 22-year-old man with a traumatic injury to the left upper limb was referred for further management from a peripheral hospital following primary and secondary surveys. On arrival to our institute he was observed to have pain disproportionate to expectation and worse pain on passive extension of his wrist.

## QUESTIONS

1. **What is compartment syndrome and what are the causes**
2. **What is the anatomy of the compartments of the forearm and hand?**
3. **What are the symptoms and signs of compartment syndrome?**
4. **What are the treatment options?**

## DISCUSSION

The first medical description linking compartment injury with ischaemia was reported by Volkmann during investigations into posttraumatic contracture.^1^ Compartment syndrome arises due to a decrease in size or an increase in volume of the anatomic components within an enclosed fascial envelope leading to increased interstitial pressure, ischaemia, and eventual muscle/nerve necrosis.tiologically, compartment syndrome can result from numerous causative factors including fractures (31%), penetrating injuries—stabbings/gunshot wounds (15%), recreational drug use (10%), crush injuries (8%), intravenous infiltration injuries (8%), and snake envenomation (6%).^2^^-^5

The boundaries of the compartments of the forearm are delineated by the lacertus fibrosus and pronator teres proximally and the carpal tunnel distally. There are 5 compartments that are interconnected. These include the superficial volar (pronator teres, palmaris longus, flexor digitorum superficialis, flexor carpi radialis, flexor carpi ulnaris), deep volar (flexor digitorum profundus, flexor pollicis longus, pronator quadratus), superficial dorsal (extensor digitorum communis, extensor carpi ulnaris, extensor digiti minimi), deep dorsal (abductor pollicis longus, extensor pollicis brevis, extensor pollicis longus, extensor indicis, supinator), and the mobile wad (brachioradialis, extensor carpi radialis longus, extensor carpi radialis brevis). The hand contains 10 compartments that are formed by the fascial enclosure of the dorsal interossei (x4), palmar interossei (x3), adductor pollicis, the thenar musculature (abductor pollicis brevis, flexor pollicis brevis, and opponens pollicis), and hypothenar musculature (abductor digiti minimi, flexor digitorum minimi, and opponens digiti minimi). The digits despite lacking muscle can also undergo increased pressures due to restrictions caused by Cleland's and Grayson's ligaments and also due to the adherence at the flexor creases.^3^^-^5

Compartment syndrome is essentially a clinical diagnosis based on history, examination, and a high index of suspicion. Diagnosis may be aided with measurement of compartment pressures with less than 30 mm Hg difference between diastolic blood pressure and the affected compartment being suggestive of compartment syndrome.^6^ Patients may present with the “P” findings of pain (disproportionate to the injury sustained or on passive extension), pallor, paraesthesia, paralysis, pulselessness, palpable fullness, and poikilothermia.^7^ With the exception of pain, however, all are relatively late findings. Nonsurgical treatment is limited to those with borderline findings and uncertain diagnosis. Initially all occlusive dressings or cast should be removed. The affected limb should then be monitored closely with serial physical examinations and pressure measurements. The injured limb should be kept at heart level during this period.^3^ Definitive management involves rapid surgical fasciotomy of the involved compartments typically commencing with the volar forearm compartment. A variety of surgical incisions are available. A standard volar incision begins 1 cm proximal and 2 cm lateral to the medial epicondyle, crossing obliquely across the antecubital fossa and over the volar aspect of the mobile wad. The incision then curves in a medial direction, reaching the midline at the junction of the middle and distal third of the forearm. The incision is then continued ulnar to palmaris longus and crosses the wrist at an angle before extending into the mid-palm for a concurrent carpal tunnel release.^3^^-^5^,^^8^ The musculature and nerves are inspected with resection of any nonviable muscle. The dorsal compartments, the mobile wad, and the compartments of the hand are then assessed to determine whether or not these require decompression. The mobile wad can be accessed via the volar incision if required. The dorsal compartments can be decompressed with a separate incision that commences 2 cm distal to the lateral epicondyle and extends distally to the wrist.^3^^,^^4^^,^^8^ Once all appropriate compartments are released, the musculature is then reassessed for viability. The incisions are left open for staged closure with or without skin grafting after the acute period. After appropriate dressings, the hand and wrist are splinted in a position of safety.^3^^-^5^,^^8^

Acute compartment syndrome is a serious surgical condition that requires careful diagnosis and rapid treatment to prevent disastrous sequelae resulting from delayed action. It is essential to have a high index of suspicion with any patient that presents with an injury to the upper limb causing pain disproportionate to what is expected.

## Figures and Tables

**Figure 1:**
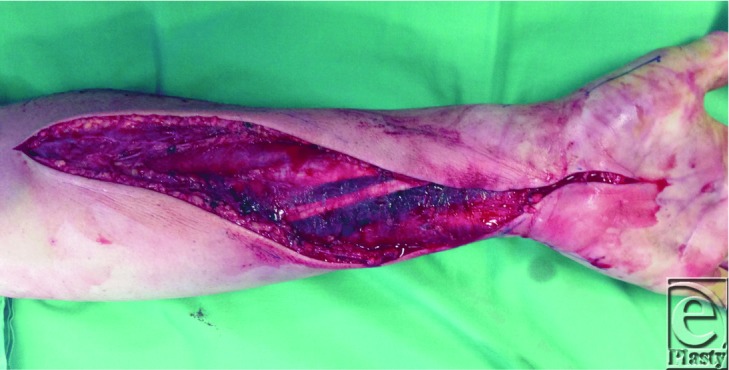
Volar incision of forearm incorporating a carpal tunnel release.

**Figure 2:**
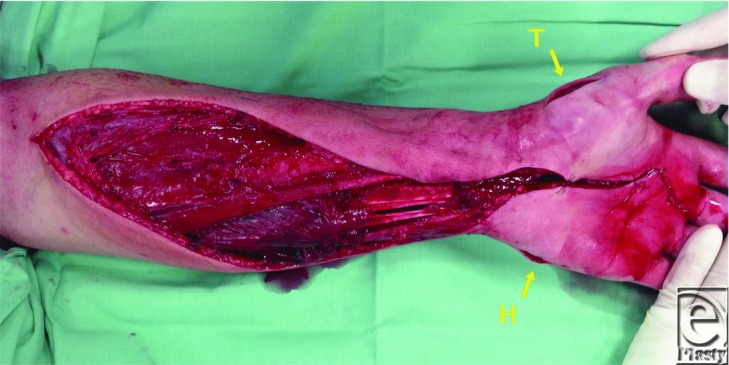
Incisions for the release of the thenar (T) and hypothenar (H) compartments.

**Figure 3:**
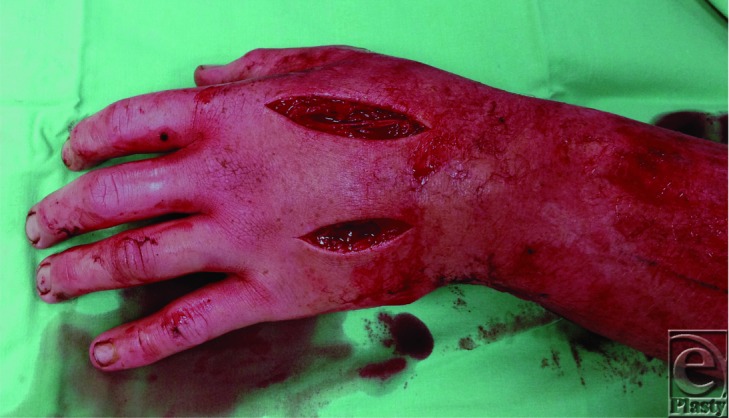
Dorsal hand incisions to access the interossei and the adductor pollicis compartments.

**Figure 4:**
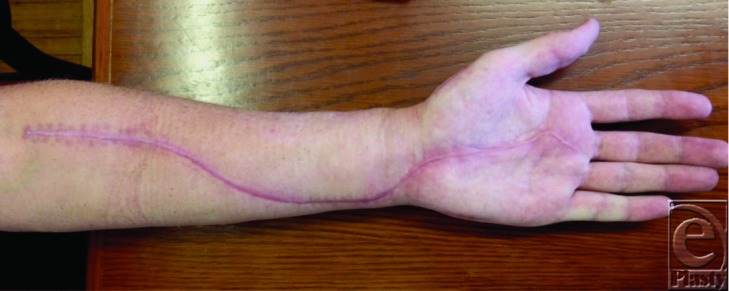
Six-month postoperative follow-up.

## References

[1] Volkmann R (1881). Die ischaemischen muskellahmungen und kontrakturen. Centralbl Chir Leipz.

[2] Matsen FA, Clawson DK (1975). Compartment syndromes: a unified concept. Clin Orthop Relat Res.

[3] Kalyani BS, Fisher BE, Roberts CS, Giannoudis PV (2011). Compartment syndrome of the forearm: a systematic review. J Hand Surg Am.

[4] Seiler JG, Olvey SP (2003). Compartment syndromes of the hand and forearm. J Hand Surg Am.

[5] Friedrich JB, Shin AY (2007). Management of forearm compartment syndrome. Hand Clin.

[6] McQueen MM, Court-Brown CM (1996). Compartment monitoring in tibial fractures: the pressure threshold for decompression. J Bone Joint Surg.

[7] Griffiths DLL (1940). Volkmann's ischaemic contracture. Br J Surg.

[8] Gelberman RH, Garlin SR, Hergenroeder PT, Mubarak SJ, Menon J (1981). Compartment syndromes of the forearm: diagnosis and treatment. Clin Orthop Relat Res.

